# Supplementary material for: Giant c-axis nonlinear anomalous Hall effect in Td-MoTe2 and WTe2
Source: Nat Commun. 2021 Apr 6;12:2049. doi: 10.1038/s41467-021-22343-5 (PMC8024290; doi:10.1038/s41467-021-22343-5)
Supplement: Supplementary file 1 — Supplementary Information [file 41467_2021_22343_MOESM1_ESM.pdf]

Supplementary Information for

Giant *c*-axis nonlinear anomalous Hall effect in T_d-$MoTe_2$ and WTe_2

Archana Tiwari[1], Fangchu Chen[1], Shazhou Zhong[1], Elizabeth Drueke[2], Jahyun Koo[3], Austin Kaczmarek[2], Cong Xiao[4], Jingjing Gao[5], Xuan Luo[5], Qian Niu[4], Yuping Sun[5,6,7], Binghai Yan[3], Liuyan Zhao[2], and Adam W. Tsen[1*]

[1]Institute for Quantum Computing, Department of Physics and Astronomy, and Department of Chemistry, University of Waterloo, Waterloo, Ontario N2L 3G1, Canada

[2]Department of Physics, University of Michigan, Ann Arbor, Michigan 48109, USA

[3]Department of Condensed Matter Physics, Weizmann Institute of Science, Rehovot 7610001, Israel

[4]Department of Physics, The University of Texas at Austin, Austin, Texas 78712, USA

[5]Key Laboratory of Materials Physics, Institute of Solid State Physics, Chinese Academy of Sciences, Hefei, 230031, China

[6]Anhui Province Key Laboratory of Condensed Matter Physics at Extreme Conditions, High Magnetic Field Laboratory, Chinese Academy of Sciences, Hefei, 230031, China

[7]Collaborative Innovation Center of Advanced Microstructures, Nanjing University, Nanjing, 210093, China

*Correspondence to: awtsen@uwaterloo.ca

Supplementary Note 1: SHG Identification of Crystal Axes

Second harmonic generation (SHG) is a nonlinear optical process in which the frequency of the incoming light is doubled through interactions with a crystal structure. In materials which do not possess a center of inversion symmetry, such as T_d-$MoTe_2$, the leading-order contribution to the SHG is the process of electric-dipole (ED) radiation, which can be expressed as:

$$P_i(2\omega) = \chi_{ijk}^{ED} E_j(\omega) E_k(\omega),$$

where $P_i(2\omega)$ is the polarization of the SHG field, $E_{j,k}(\omega)$ are components of the incoming fundamental electric field, and χ_{ijk}^{ED} is the SHG susceptibility tensor of the electric-dipole approximation. Importantly, the general form of χ^{ED} is determined by the symmetries of the crystal.

The rotational anisotropy of the SHG (RA-SHG) was measured using an experimental setup shown in Supplementary Fig. 1. The intensity of the reflected SHG, $I^{2\omega}$, was measured as a function of the angle φ between the incident polarization and the *x*-axis in the lab coordinate frame. The incident fundamental and the reflected SHG polarizations can be selected to be either parallel or crossed, forming the two polarization channels of the RA-SHG measurements.

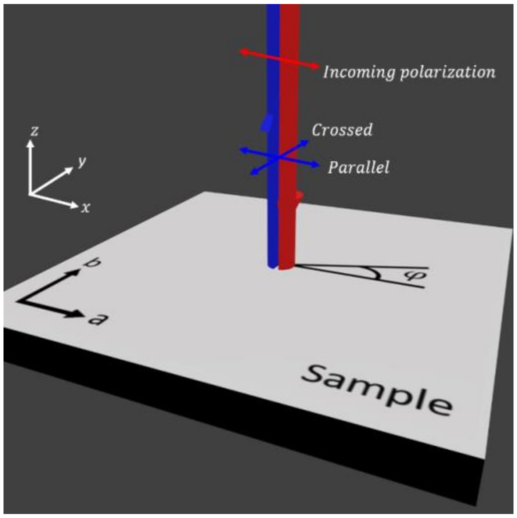

Supplementary Fig. 1. The RA-SHG setup. Incoming light at 800nm is incident normal to the sample surface and the intensity of the reflected SHG light (400nm) is measured as a function of the angle of rotation of the polarization about the *c*-axis of the crystal. There are two unique polarization channels in this normal incidence geometry – parallel and crossed. Here, the *a* and *b* axes of the crystal are indicated as well as the lab-frame coordinates *xyz*.

The space group of T_d-$MoTe_2$ bulk crystals is known to be the non-centrosymmetric $Pmn2_1$ with a mirror plane normal to the *a*-axis, a glide plane perpendicular to the *b*-axis, and a C_2 screw axis along the *c*-axis[1]. Above a critical temperature of T_c = 250K, the crystal undergoes a structural phase transition if the thickness of the layers is above ~12nm to 1T'-$MoTe_2$ in which the C_2 axis becomes an in-plane screw axis (along the *a*-axis) and the glide plane disappears (centrosymmetric space group $P2_1/m$)[2]. For samples with thickness below ~12nm, the T_d structure obeys space group

Pm with a mirror plane normal to the *a*-axis because the glide plane is not preserved[3]. In such thin samples, this crystal structure is maintained above 250K[4].

At the surface of bulk or thick flake of T_d-$MoTe_2$, the out-of-plane 2-fold screw axis is no longer present because of the lack of translational symmetry along the surface normal direction. As a result, the surface point group for T_d-$MoTe_2$ is *m*, where a single mirror plane is normal to the a-axis. Optical SHG under the ED approximation is extremely sensitive to the surface contribution, and therefore its corresponding susceptibility tensor $\chi^{(2)}_{MoTe_2}$ should be derived using the surface point group *m* (shown below, where *x*, *y*, and *z* are equivalent to *a*, *b*, and *c*, respectively). The rotational anisotropy (RA) of ED SHG can be further computed based on $\chi^{(2)}_{MoTe_2}$.

$$\chi^{(2)}_{MoTe_2} = \begin{pmatrix} \begin{pmatrix} 0 \\ \chi^{MoTe_2}_{yxx} \\ \chi^{MoTe_2}_{zxx} \end{pmatrix} & \begin{pmatrix} \chi^{MoTe_2}_{yxx} \\ 0 \\ 0 \end{pmatrix} & \begin{pmatrix} \chi^{MoTe_2}_{zxx} \\ 0 \\ 0 \end{pmatrix} \\ \begin{pmatrix} \chi^{MoTe_2}_{yxx} \\ 0 \\ 0 \end{pmatrix} & \begin{pmatrix} 0 \\ \chi^{MoTe_2}_{yyy} \\ \chi^{MoTe_2}_{zyy} \end{pmatrix} & \begin{pmatrix} 0 \\ \chi^{MoTe_2}_{zyy} \\ \chi^{MoTe_2}_{zzy} \end{pmatrix} \\ \begin{pmatrix} \chi^{MoTe_2}_{zxx} \\ 0 \\ 0 \end{pmatrix} & \begin{pmatrix} 0 \\ \chi^{MoTe_2}_{zyy} \\ \chi^{MoTe_2}_{zzy} \end{pmatrix} & \begin{pmatrix} 0 \\ \chi^{MoTe_2}_{zzy} \\ \chi^{MoTe_2}_{zzz} \end{pmatrix} \end{pmatrix}.$$

We can use this to simulate functional forms for the ED RA SHG intensity together with

$$I^{2\omega}(\varphi) = \left|A\chi^{ED}_{ijk}(\varphi)\hat{e}_j(\omega)\hat{e}_k(\omega)\right|^2 I^{\omega}I^{\omega},$$

where A is a constant determined by the experimental geometry and $\hat{e}_{j,k}(\omega)$ are components of the polarization of the incoming fundamental light. Doing this, we achieve the following functional forms:

$$I^{2\omega}_{MoTe_2,parallel} = \left(3\chi^{MoTe_2}_{yxx}\cos^3(\alpha-\varphi)\sin(\alpha-\varphi) + \chi^{MoTe_2}_{yyy}\sin^3(\alpha-\varphi)\right)^2$$

$$I^{2\omega}_{MoTe_2,crossed} = \frac{1}{4}\cos^2(\alpha-\varphi)\left(\chi^{MoTe_2}_{yxx} - \chi^{MoTe_2}_{yyy} + \left(\chi^{MoTe_2}_{yyy} - 3\chi^{MoTe_2}_{yxx}\right)\cos\left(2(\alpha-\varphi)\right)\right)^2,$$

where α defines the rotation angle of the sample's mirror plane from the mirror plane normal to the *a*-axis used to derive the model. It is through this α that the mirror plane orientation of the device is determined.

In addition to contributions from the $MoTe_2$ flakes, it was found that the h-BN flakes encapsulating the $MoTe_2$ also contributed to the detected SHG. To account for this, the models for the two polarization channels were amended to include contributions from the h-BN by coherently summing the SHG electric fields from both materials. The model was derived assuming that the mirror plane of the h-BN was rotated from the *y*-axis by an angle δ and included a complex phase γ between the fields from the two materials. Illustrations of this coherent summing procedure are shown in Supplementary Fig. 2.

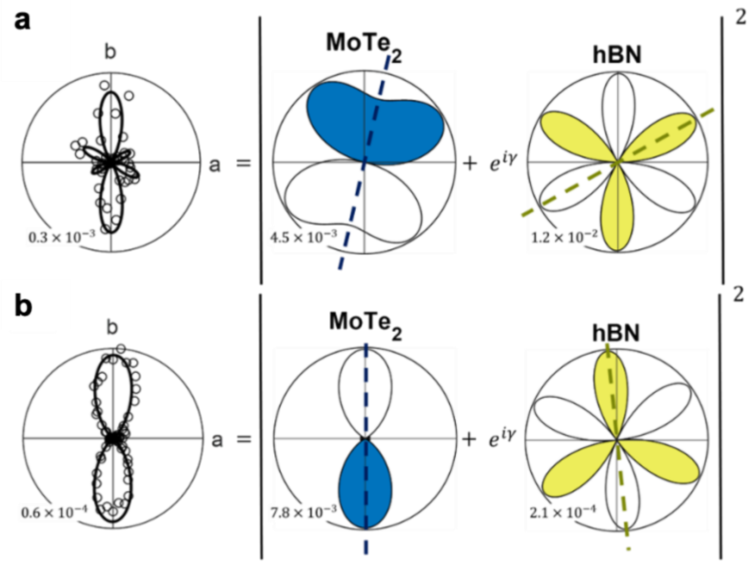

Supplementary Fig. 2. RA-SHG patterns for $MoTe_2$ devices. Patterns for sample thickness **a,** 127nm and **b,** 47nm shown with the derived fits for the T_d-$MoTe_2$ and h-BN contributions as they are coherently summed. The mirror planes extracted from the fits using the α and δ parameters are shown by the dashed blue and yellow lines, respectively. The shading indicates positive and negative values of the E-fields. Scaling is in units of counts per second on the detector. The shown examples here are for the parallel channel only for each device.

Few layer h-BN is well-known to exist in the D_{3h} point group[5], yielding the SHG susceptibility tensor:

$$\chi^{(2)}_{hBN} = \begin{pmatrix} \begin{pmatrix} 0 \\ -\chi^{hBN}_{yyy} \\ 0 \end{pmatrix} & \begin{pmatrix} -\chi^{hBN}_{yyy} \\ 0 \\ 0 \end{pmatrix} & \begin{pmatrix} 0 \\ 0 \\ 0 \end{pmatrix} \\ \begin{pmatrix} -\chi^{hBN}_{yyy} \\ 0 \\ 0 \end{pmatrix} & \begin{pmatrix} 0 \\ \chi^{hBN}_{yyy} \\ 0 \end{pmatrix} & \begin{pmatrix} 0 \\ 0 \\ 0 \end{pmatrix} \\ \begin{pmatrix} 0 \\ 0 \\ 0 \end{pmatrix} & \begin{pmatrix} 0 \\ 0 \\ 0 \end{pmatrix} & \begin{pmatrix} 0 \\ 0 \\ 0 \end{pmatrix} \end{pmatrix}.$$

Coherently summing the fields from the T_d-$MoTe_2$ and the h-BN using the procedure outlined above yields the following functional forms:

$$\begin{aligned} I^{2\omega}_{P_{in}-P_{out}}(2\omega) = {}& \left(3\chi^{MoTe_2}_{yxx}\, cos^2(\alpha-\varphi)\, sin(\alpha-\varphi) + \chi^{MoTe_2}_{yyy}\, sin^3(\alpha-\varphi)\right)^2 \\ & - 2\chi^{hBN}_{yyy}\, cos(\gamma)\, sin(\alpha-\varphi)\left(3\chi^{MoTe_2}_{yxx}\, cos^2(\alpha-\varphi) \right. \\ & \left. + \chi^{MoTe_2}_{yyy}\, sin^2(\alpha-\varphi)\right) sin\big(3(\delta-\varphi)\big) + {\chi^{hBN}_{yyy}}^2\, sin^2\big(3(\delta-\varphi)\big), \end{aligned}$$

$$\begin{aligned} I^{2\omega}_{P_{in}-S_{out}}(2\omega) = {}& \frac{1}{4}\Big(cos^2(\alpha-\varphi)\left(\chi^{MoTe_2}_{yxx} - \chi^{MoTe_2}_{yyy} + \left(-3\chi^{MoTe_2}_{yxx} + \chi^{MoTe_2}_{yyy}\right) cos^2\big(2(\alpha-\varphi)\big)\right) \\ & + 4\chi^{hBN}_{yyy}\, cos(\gamma)\, cos(\alpha-\varphi)\left(\chi^{MoTe_2}_{yxx} - \chi^{MoTe_2}_{yyy} \right. \\ & \left. + \left(-3\chi^{MoTe_2}_{yxx} + \chi^{MoTe_2}_{yyy}\right) cos\big(2(\alpha-\varphi)\big)\right) cos\big(3(\delta-\varphi)\big) \\ & + 4\chi^{hBN}_{yyy}\, cos^2\big(3(\delta-\varphi)\big)\Big). \end{aligned}$$

Our fittings thus extract the susceptibility tensor elements as well as the angles α, δ and the phase γ.

Supplementary Note 2: Measurement of in-plane NLAHE in 2D $MoTe_2$

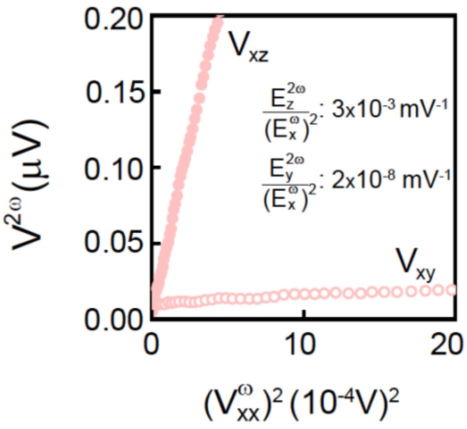

Supplementary Fig. 3. Comparison of in-plane and out-of-plane Hall responses. Second harmonic V_{xz} and V_{xy} measured in 9nm-thick sample for *I* || *a* at 2K shows stronger out-of-plane NLAHE strength.

Supplementary Note 3: Dependence of NLAHE on Frequency and Current Direction

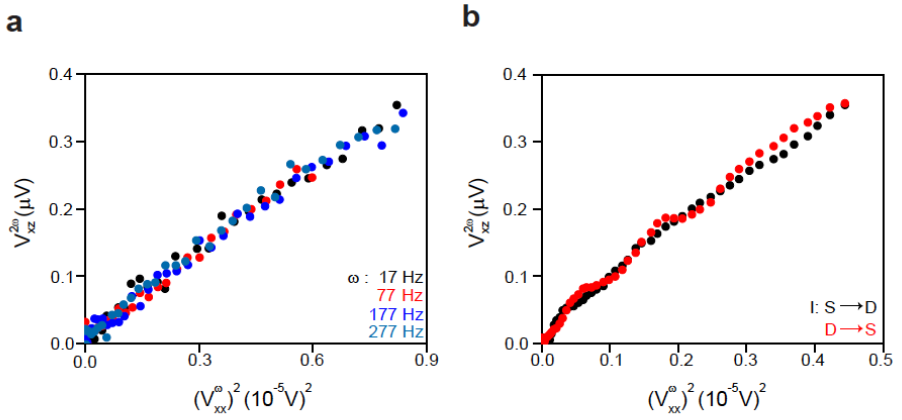

Supplementary Fig. 4. Fidelity of NLAHE signal. The *c*-axis NLAHE does not substantially change with **a,** changing first harmonic frequency or **b,** exchanging the current leads.

Supplementary Note 4: Finite Element Method Simulations of Current and Potential Distributions

In order to account for the circular electrode geometry and unique shape of each $MoTe_2$ flake, we have performed simulations using the finite element method (FEM) to determine the precise current and potential distribution for each individual device. We obtain the in-plane conductivities by matching the simulated potential difference with that measured experimentally between the particular voltage leads V_{xx} for a given directional current bias. We then extract the local electric field E_x at the vertical contact/junction for that bias. An example simulation is shown below for the 70nm device.

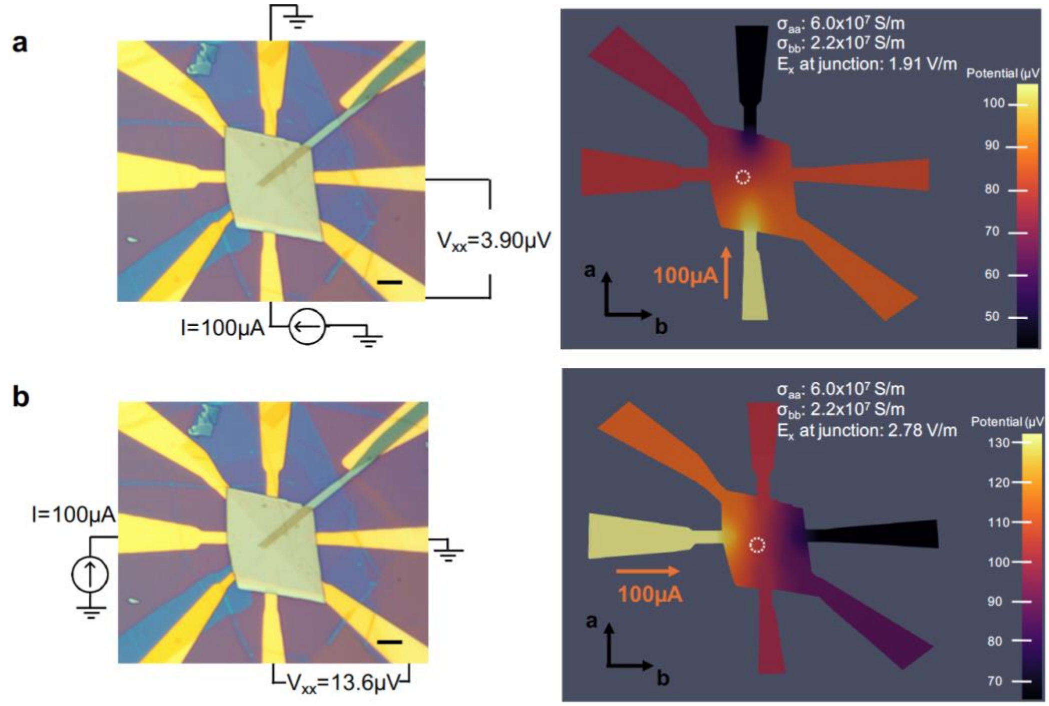

Supplementary Fig. 5. Representative FEM simulations taken of 70nm-thick device. a, Left: optical image of device and bias/measurement circuit (I || a). Right: simulated potential distribution corresponding to the circuit conditions used to extract the in-plane conductivities and longitudinal electric field locally at the vertical contacts used to measure the c-axis NLAHE. **b,** Same but for I || b. Scale bars are 5μm.

Supplementary Note 5: Comparison with WTe_2

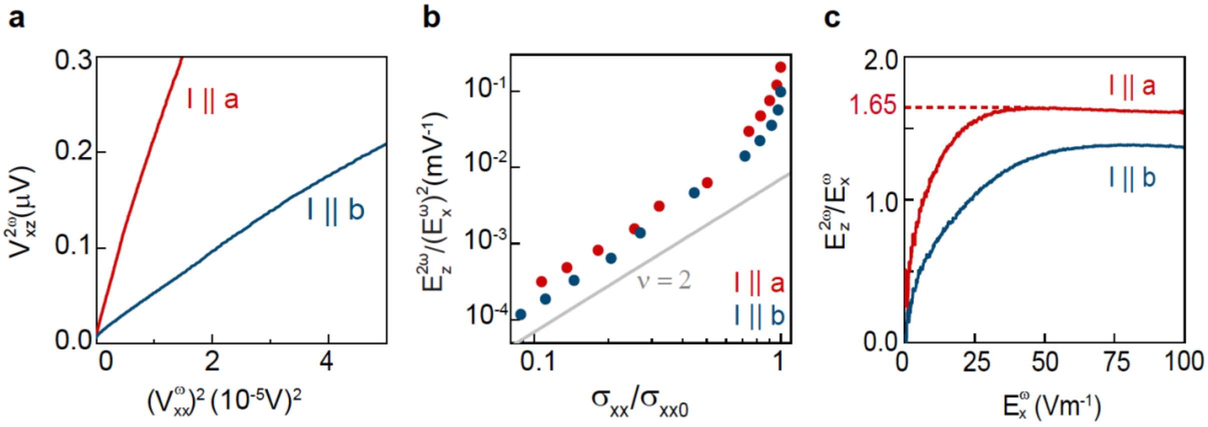

Supplementary Fig. 6. Measurement of *c*-axis NLAHE in bulk-like WTe_2. a, Second harmonic V_{xz} vs. first harmonic V_{xx}^2 for $I \parallel a$ and $I \parallel b$ in 130-nm-thick sample at 2K. **b,** NLAHE strength $E_z^{2\omega}/(E_x^{\omega})^2$ vs. σ_{xx}/σ_{xx0} for $I \parallel a$ and $I \parallel b$. **c,** Hall angle $E_z^{2\omega}/E_x^{\omega}$ vs E_x^{ω} for $I \parallel a$ and $I \parallel b$. Peak NLAHE strength and Hall angle is less than but comparable to 127-nm-thick $MoTe_2$.

Supplementary Note 6: $MoTe_2$ Hall Bar Devices

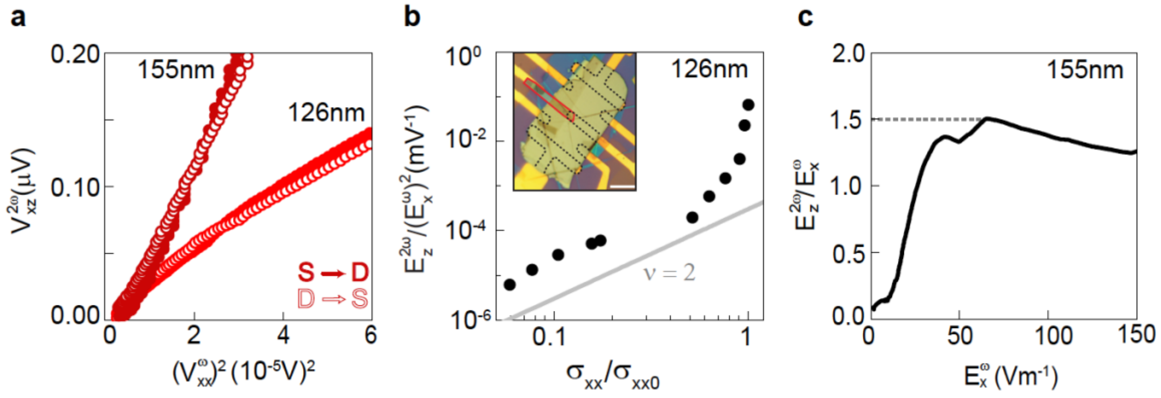

Supplementary Fig. 7. Measurement of *c*-axis NLAHE in $MoTe_2$ Hall bars. a, Second harmonic V_{xz} vs. first harmonic V_{xx}^2 for $I \parallel a$ in 155-nm- and 126-nm-thick samples at 2K. The response does not change substantially when exchanging the current leads. **b,** NLAHE strength $E_z^{2\omega}/(E_x^{\omega})^2$ vs. σ_{xx}/σ_{xx0} for $I \parallel a$ in 126-nm-thick sample. Inset shows optical image of device with bottom (top) electrodes traced out in black (red). Scale bar is 5μm. **c,** Hall angle $E_z^{2\omega}/E_x^{\omega}$ vs E_x^{ω} for $I \parallel a$ in 155-nm-thick sample.

Supplementary Note 7: Determination of the Intrinsic Berry Curvature Contribution to the NLAHE strength

In the NLAHE, the vertical Hall current is given by $j_z^{2\omega} = \sigma_{zz}E_z^{2\omega} = 2\chi_{zxx}\,(E_x^{\omega})^2$, where the intrinsic Berry curvature dipole (D_{xy}) contribution to the nonlinear susceptibility tensor is given by: $\chi_{zxx} = -\varepsilon_{zyx}\,\frac{e^3\tau}{2\,\hbar^2(1+i\omega\tau)}D_{xy}$ [6]. ε_{zyx} stands for the third-rank Levi-Civita symbol. In the DC limit $\omega\tau \ll 1$, we have: $\frac{E_z^{2\omega}}{(E_x^{\omega})^2} = \frac{1}{\sigma_{zz}}\frac{e^3\tau}{\hbar^2}\,D_{xy}$.

For each sample, we have extracted the electron and hole densities (n, p) and mobilities (μ_n, μ_p) using a two-band model for the ordinary Hall effect and magnetoresistance[7]. Supplementary Fig. 8 shows representative data and extracted values for the 127-nm-thick device for $I \parallel a$. The electron and hole densities are nearly balanced. We can then obtain the corresponding scattering time $\tau = \frac{\mu\, m_{eff}}{e}$, where $m_{eff} \sim m_0$, the bare electron mass[7]. τ ranges between ~0.2ps (9nm) to ~1ps (127nm).

From the DFT calculations shown in Supplementary Fig. 10, $\sigma_{zz} \sim 0.6\sigma_{xx}$ for I ∥ a near the charge neutrality point, while the theoretical value for D_{xy} is 0.85[6]. From these values, we calculate an upper limit of $E_z^{2\omega}/(E_x^{\omega})^2 = 1.3 \times 10^{-7}\,\mathrm{m/V}$ across all our different samples.

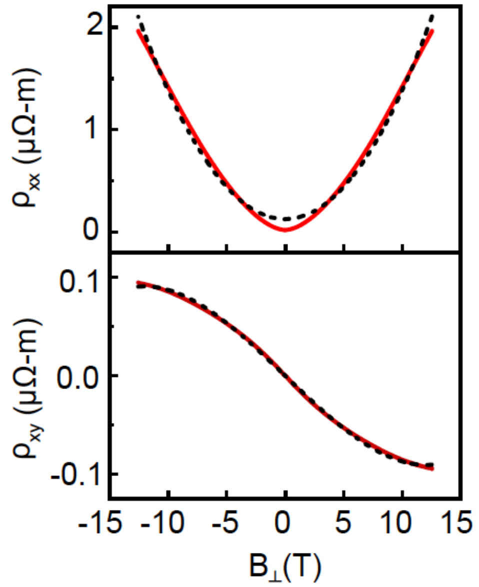

$$n = 0.77 \times 10^{26} \mathrm{m}^{-3}$$
$$p = 0.78 \times 10^{26} \mathrm{m}^{-3}$$
$$\mu_n = 0.36 \mathrm{m}^2 \mathrm{V}^{-1} \mathrm{s}^{-1}$$
$$\mu_p = 0.27 \mathrm{m}^2 \mathrm{V}^{-1} \mathrm{s}^{-1}$$

Supplementary Fig. 8. Representative magnetoresistance and Hall data. Symmetrized magnetoresistance and anti-symmetrized Hall measurements taken on 127-nm-thick sample for $I \parallel a$. Dashed lines are fits to the two-band model, yielding electron density (n), hole density (p), electron mobility (μ_n), and hole mobility (μ_p).

Supplementary Note 8: Measurement of NLAHE at Higher Bias

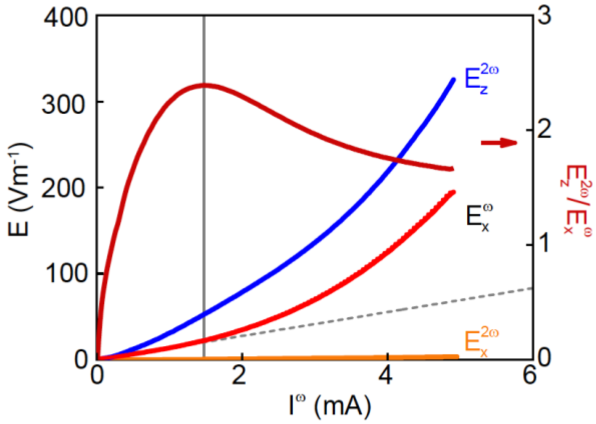

Supplementary Fig. 9. Measurement of NLAHE at higher bias. $E_x^{\omega}, E_x^{2\omega}, E_z^{2\omega}$, and Hall angle $\mathrm{E}_z^{2\omega}/\mathrm{E}_x^{\omega}$ vs. $I \parallel a$ for 127-nm-thick sample. Solid gray line marks the current at which Hall angle is maximum. Beyond this current, E_x^{ω} begins to deviate from a linear dependence (dashed gray line), likely due to sample heating, although $E_x^{2\omega}$ remains negligibly small in comparison.

Supplementary Note 9: Determination of Conductivity Anisotropy

We performed first-principles calculation to estimate the conductivity anisotropy of bulk T_d-phase $MoTe_2$. Our calculations were based on the density-functional theory (DFT) in the framework of the generalized gradient approximation with the Vienna ab-intio package[8,9]. The spin-orbit

coupling was included. The longitudinal conductivity was evaluated by the semi-classical transport theory using the Boltzmann transport equation. The lattice structure taken as $Pmn2_1$ space group with a = 3.48Å, b = 6.34Å, and c = 13.88Å.

By assuming the same relaxation time along the different crystal directions, we obtain the ratio between out-of-plane conductivity (σ_{cc}) and in-plane conductivity (σ_{aa}) with respect to the chemical potential. Near the charge neutral point (zero energy), the $\frac{\sigma_{cc}}{\sigma_{aa}}$ ~0.6. Although it is a layered structure, the c-axis conductivity is in the same order of magnitude as the in-plane conductivity.

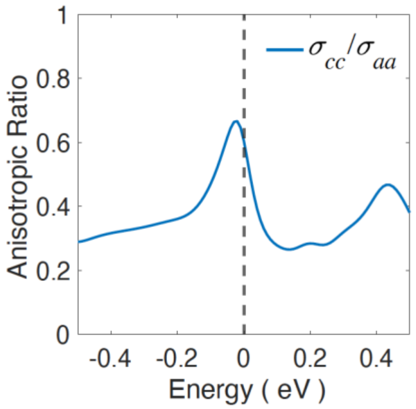

Supplementary Fig. 10. Calculation of conductivity anisotropy. Calculated ratio between the c-axis conductivity (σ_{cc}) and in-plane conductivity (σ_{aa}) of T_d-$MoTe_2$ as a function of the Fermi energy. Zero energy corresponds to the charge neutrality point where electrons and holes compensate.

We have also measured experimentally the temperature-dependent resistivity along the a and c axes of a separate bulk $MoTe_2$ crystal grown under the same conditions as those used for our devices. ρ_{cc}/ρ_{aa} ~4 across nearly the entire temperature range.

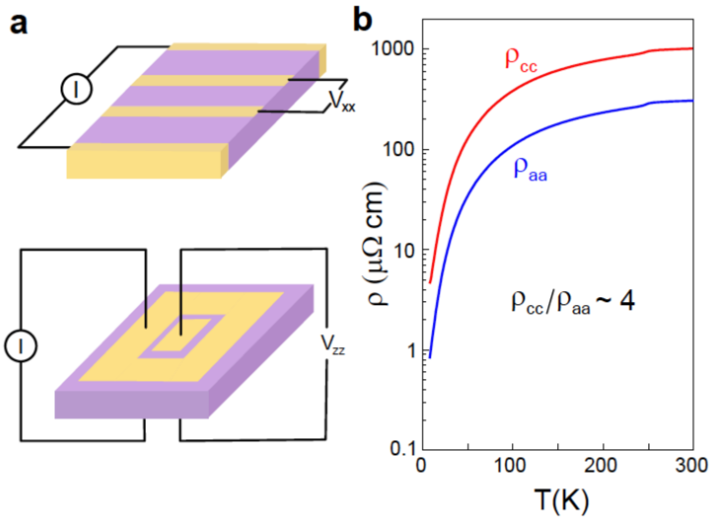

Supplementary Fig. 11. Measurement of resistivity anisotropy. a, Schematic of measurement geometry. b, Temperature-dependent resistivity for $I \parallel a$ and $I \parallel c$. Anisotropy ratio is nearly constant across the entire temperature range and is the same order as the calculated value.

Supplementary References:

1. Fei, Z. *et al.* Ferroelectric switching of a two-dimensional metal. *Nature* **560**, 336–339 (2018).
2. Xu, S. Y. *et al.* Electrically switchable Berry curvature dipole in the monolayer topological insulator WTe_2. *Nature Physics* **14**, 900–906 (2018).
3. Song, Q. *et al.* The In-Plane Anisotropy of WTe_2 Investigated by Angle-Dependent and Polarized Raman Spectroscopy. *Sci. Rep.* **6**, 1–9 (2016).
4. He, R. *et al.* Dimensionality-driven orthorhombic $MoTe_2$ at room temperature. *Phys. Rev. B* **97**, 041410(R) (2018).
5. Michel, K. H. & Verberck, B. Theory of elastic and piezoelectric effects in two-dimensional hexagonal boron nitride. *Phys. Rev. B* **80**, 224301 (2009).
6. Zhang, Y., Sun, Y. & Yan, B. Berry curvature dipole in Weyl semimetal materials: An ab initio study. *Phys. Rev. B* **97**, 041101 (2018).
7. Zhong, S. *et al.* Origin of magnetoresistance suppression in thin $\gamma-MoTe_2$. *Phys. Rev. B* **97**, 241409(R) (2018).
8. Perdew, J. P., Burke, K. & Ernzerhof, M. Generalized gradient approximation made simple. *Phys. Rev. Lett.* **77**, 3865–3868 (1996).
9. Joubert, D. From ultrasoft pseudopotentials to the projector augmented-wave method. *Phys. Rev. B* **59**, 1758–1775 (1999).
